# Development, Validation, and Visualization of A Web-Based Nomogram for Predicting the Recurrence-Free Survival Rate of Patients With Desmoid Tumors

**DOI:** 10.3389/fonc.2021.634648

**Published:** 2021-02-25

**Authors:** Haotian Liu, Kai Huang, Tao Li, Tielong Yang, Zhichao Liao, Chao Zhang, Lijie Xiang, Yong Chen, Jilong Yang

**Affiliations:** ^1^ Department of Bone and Soft Tissue Tumor, Tianjin Medical University Cancer Institute & Hospital, Tianjin, China; ^2^ National Clinical Research Center for Cancer, Key Laboratory of Cancer Prevention and Therapy, Tianjin’s Clinical Research Center for Cancer, Tianjin Medical University Cancer Institute & Hospital, Tianjin, China; ^3^ Department of Musculoskeletal Oncology, Fudan University Shanghai Cancer Center, Shanghai, China; ^4^ Department of Oncology, Shanghai Medical College, Fudan University, Shanghai, China; ^5^ Brandon Regional Hospital GME, HCA Healthcare/USF Morsani College of Medicine, Brandon, FL, United States; ^6^ Department of Bone and Soft-Tissue Tumor, Institute of Cancer and Basic Medicine, Chinese Academy of Sciences, Cancer Hospital of the University of Chinese Academy of Sciences, Zhejiang Cancer Hospital, Hangzhou, China

**Keywords:** desmoid tumor, recurrence, prediction, the web-based nomogram, tumor number, validation

## Abstract

**Background:**

Surgery is an important treatment option for desmoid tumor (DT) patients, but how to decrease and predict the high recurrence rate remains a major challenge.

**Methods:**

Desmoid tumor patients diagnosed and treated at Tianjin Cancer Institute & Hospital were included, and a web-based nomogram was constructed by screening the recurrence-related risk factors using Cox regression analysis. External validation was conducted with data from the Fudan University Shanghai Cancer Center.

**Results:**

A total of 385 patients were identified. Finally, after excluding patients without surgery, patients who were lost to follow-up, and patients without complete resection, a total of 267 patients were included in the nomogram construction. Among these patients, 53 experienced recurrence, with a recurrence rate of 19.85%. The 3-year and 5-year recurrence-free survival (RFS) rates were 82.5% and 78%, respectively. Age, tumor diameter, admission status, location, and tumor number were correlated with recurrence in univariate Cox analysis. In multivariate Cox analysis, only age, tumor diameter and tumor number were independent risk factors for recurrence and were then used to construct a web-based nomogram to predict recurrence. The concordance index (C-index) of the nomogram was 0.718, and the areas under the curves (AUCs) of the 3-year and 5-year receiver operating characteristic (ROC) curves were 0.751 and 0.761, respectively. In the external validation set, the C-index was 0.706, and the AUCs of the 3-year and 5-year ROC curves are 0.788 and 0.794, respectively.

**Conclusions:**

Age, tumor diameter, and tumor number were independent predictors of recurrence for DTs, and a web-based nomogram containing these three predictors could accurately predict RFS (https://stepforward.shinyapps.io/Desmoidtumor/).

## Introduction

Desmoid tumors (DTs), also known as aggressive fibromatosis and desmoid-type fibromatosis, have an incidence of approximately 5 per 1 million persons and are characterized by no metastasis, but local progression and recurrence can occur (1, 2). In general, DTs can be divided into sporadic and familial adenomatous polyposis (FAP)-related DTs. Sporadic DTs are associated with a mutation in the CTNNB1 gene and are usually located in the abdominal wall or outside the abdomen (3). DTs on the abdominal wall are most common in women within one year after pregnancy or childbirth. FAP-related DTs, which are associated with mutations in the APC gene, usually occur in the intra-abdominal region, including the mesentery, retroperitoneal area, or pelvis, and can be very large (2, 4).

DTs can occur at any age; the typical age of onset is 15–60 years, with a peak of onset at approximately 30 years (5, 6). The pathogenesis of DTs is related to many factors, including trauma, hormones, and heredity. Trauma, including surgery, may cause DTs (7). It was found that the incidence was higher in females, a significant number of women had a recent pregnancy history at the time of diagnosis, and the risk of progression during pregnancy was higher, which suggests that the disease is related to hormones (8). Heredity mainly refers to FAP patients being prone to developing DTs. It has been reported that patients with FAP are 1,000 times more likely to develop DTs than those without FAP (7). In addition, 21%–31% of FAP patients may develop DTs (6, 7). The natural history and clinical symptoms of desmoid-type fibromatosis vary significantly. In some cases, the tumor may be stable for a long time without obvious clinical symptoms, while in other cases, it may rapidly increase in size. Intra-abdominal DTs can cause intestinal obstruction (9). DTs located in the extremities are generally not life-threatening conditions that would cause a patient’s death, but they can cause patients to have pain, restricted mobility, and loss of function (9). In some studies, one of the problems faced by patients with extremity tumors is poor prognosis; that is, the probability of recurrence after surgical resection is higher than in other locations, and some patients may even face the risk of amputation (10, 11). Therefore, care must be taken in the management of patients with extremity tumors.

With an improved understanding of the natural course of DTs, changes in the treatment of DTs have also emerged. According to the latest treatment guidelines, the preferred treatment for DTs is no longer surgery or other treatments but close conservative observation (12, 13). There are a number of patients whose tumors do not progress during observation (14). However, surgery remains an important treatment option when patients experience pain or pressure or other clinical symptoms, and chemotherapy, hormone therapy, and targeted therapy may also be considered when the tumor cannot be resected or the surgery would lead to serious functional loss (15, 16). Radiotherapy can also treat DT. James et al. suggested that radiotherapy is recommended for unresectable or recurrent DT (17). According to the latest NCCN guidelines (2020.V2), radiotherapy is not recommended for intra-abdominal DT. For patients with superficial trunk or extremity DT, definitive radiotherapy can be used as first-line or second-line treatment. On the other hand, radiotherapy may also induce secondary malignant tumors. Currently, one of the major challenges in surgical treatment is the high postoperative recurrence rate, which in some studies is as high as 60% (18). To date, recognized risk factors for recurrence include age, site, tumor size, and genetic mutations. Patients with positive expression of Ki-67 and a high signal on T2-weighted MRI have a higher probability of recurrence after surgery (19). In addition, higher PARP-1 (a DNA repairing enzyme) expression is also associated with earlier recurrence of DTs (20). In 2013, Crago et al. constructed a nomogram, which included age, location and tumor diameter based on these recurrence factors, that can roughly predict the probability of recurrence in DT patients. In addition, whether postoperative recurrence of DTs is related to other factors remains to be explored. For example, tumor number is rarely mentioned in previous studies because of the rarity of multiple tumors. In addition, the development of a tool that allows both doctors and patients to easily predict the probability of postoperative recurrence and to provide patients with a more reasonable treatment plan are also promising research directions. In the present study, we constructed a new web-based nomogram that shows good discrimination and calibration in both the development population and validation population and provides prediction probabilities that are more accurate, convenient and visual.

## Methods

### Data Collection

After obtaining permission from the Tianjin Medical University Cancer Institute & Hospital ethics committee, the medical records of 385 patients diagnosed with DT pathologically were consecutively reviewed from January 2007 to June 2019. The information of these patients is identified from the medical record database. The following clinical and demographic variables were recorded: age, sex, location, pathological diagnosis, treatment methods, tumor diameter, tumor number, and resection margin status.

**Figure 1:**
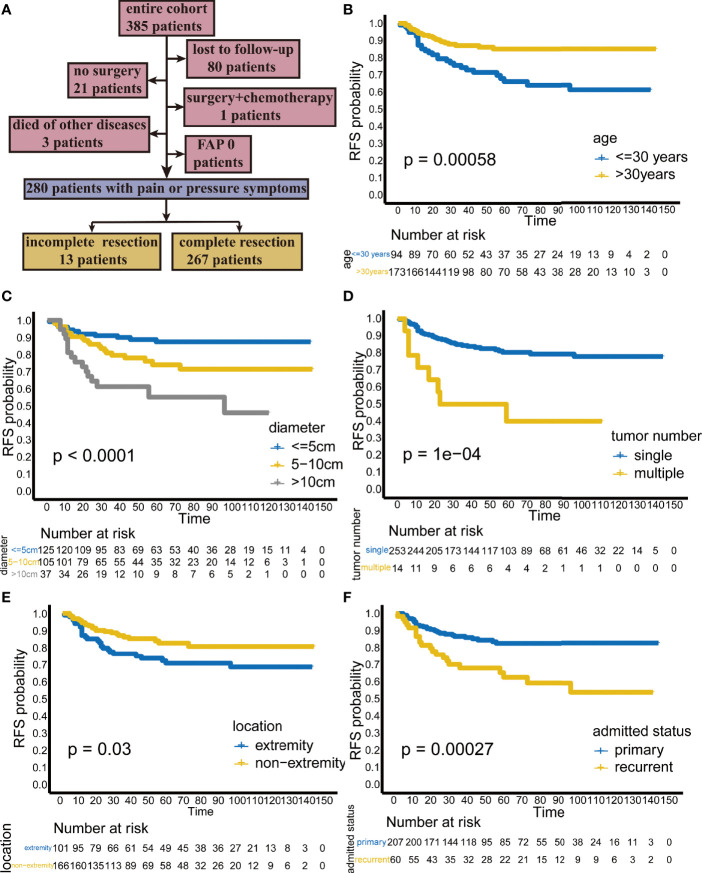
**(A)** Flow chart of patient selection. Kaplan-Meier analysis showing the recurrence-free survival (RFS) rate stratified by **(B)** age, **(C)** diameter, **(D)** tumor number, **(E)** location, and **(F)** admission status.

Patients were followed up every three months during the first year after surgery. Patients were followed up every 6 months during the second year after surgery. Follow-up once a year occurred thereafter. After five years of follow-up, patients were admitted to the hospital only if they had clinical symptoms. Follow-up was performed mainly by ultrasound or MRI. The dates of surgery and recurrence were recorded. Finally, eighty patients were lost to follow-up, 21 patients did not undergo surgery, 3 patients died of other diseases (unknown specific time of death), 1 patient underwent surgery + chemotherapy, and none were diagnosed with FAP. These patients were excluded from the final analysis. The remaining 280 patients were treated surgically because of symptoms of pain or compression, and it was the purpose of this study to analyze the prognosis of these patients who received surgery. Of these, 13 patients received incomplete resection and 267 patients received complete resection (**Figure 1A**). Complete resection is defined as no residual lesion macroscopically or microscopically (R0 margin). For patients with multiple tumors, complete resection is defined as removal of all lesions. Because we explored the predictors of recurrence-free survival (RFS) after complete resection, the analysis was performed on the 267 patients who underwent complete resection at our hospital. After reviewing the pathology reports of these patients, we found that the margin status of these 267 patients were all negative (R0).

The external validation dataset included 143 patients who underwent R0 resection at Fudan University Shanghai Cancer Hospital. The margin status was also negative in these 143 patients.

### Data Analysis

Postoperative recurrence was the primary end point and defined as the presence of clinically visible or radiologically assessable lesions. RFS is defined as the time from surgery to recurrence. Site was classified into the trunk, extremity, head and neck, and intra-abdominal region. Age and tumor diameter were included as categorical variables in univariate Cox analysis, multivariate Cox analysis and the construction of the nomogram, where age was divided into two groups (<=30 years and >30 years), and tumor diameter was obtained from the pathology reports and classified into three groups (<=5 cm, 5–10 cm and >10 cm).

The chi-square test and the Fisher exact test were used to analyze categorical data. The Kruskal-Wallis test was used to analyze continuous data with nonnormal distributions. The Kaplan-Meier (KM) method was used to calculate RFS curves. The log-rank test was performed to assess the significance of differences between RFS curves. Univariate Cox and multivariate Cox analysis were used to screen risk factors related to recurrence. Cox analysis results were presented as the hazard ratio (HR) and 95% confidence interval (CI). Among them, the variables with p<0.05 in the univariate Cox analysis were included in the multivariate Cox regression for further analysis. In KM analysis and Cox analysis, all p values reported are two-sided, and p<0.05 was considered statistically significant. IBM SPSS 22 was used for data analysis.

The discrimination of this web-based nomogram was evaluated by the concordance index (C-index) and the receiver operating characteristic (ROC) curve. The calibration was evaluated by the calibration plot.

## Results

### Patient Characteristics

The patient demographics and tumor characteristics are shown in **Table 1**. Of the 267 patients consulted at our hospital, 207 (77.5%) had primary lesions, and 60 (22.5%) had recurrent lesions. There were 80 male patients (30%) and 187 female patients (70%), suggesting that females were more affected. The median age of the patients was 35 years (range, 1–87 years). The most common site was the trunk (n = 170, 63.7%), followed by the extremities (n = 69, 25.8%), intra-abdominal region (n =16, 6.0%), and head/neck (n =12, 4.5%). Most patients had a single tumor (253 patients (94.8%)), while only 14 patients (5.2%) had multiple tumors.

**Table 1:** Patient demographics and tumor characteristics in the development dataset.

	Overall	Primary	Recurrent	P
**Patients**	267	207(77.5)	60(22.5)	
**Gender (%)**				0.26
Male	80 (30.0)	58 (28.0)	22 (36.7)	
Female	187 (70.0)	149 (72.0)	38 (63.3)	
**Age**				
**Median [range]**	35.00 [1, 87]	36.00 [2,87]	32.50 [1,80]	0.116
<=30 years	94 (35.2)	67 (32.4)	27 (45.0)	0.099
>30 years	173 (64.8)	140 (67.6)	33 (55.0)	
**Site (%)**				0.002
Trunk	170 (63.7)	136 (65.7)	34 (56.7)	
Extremity	69 (25.8)	44 (21.3)	25 (41.7)	
Head/neck	12 (4.5)	11 (5.3)	1 (1.7)	
Intra-abdominal	16 (6.0)	16 (7.7)	0 (0.0)	
**Disease number (%)**				0.027
Single	253 (94.8)	200 (96.6)	53 (88.3)	
Multiple	14 (5.2)	7 (3.4)	7 (11.7)	
**Diameter**				
**Median [range]**	6.00 [1.00,20.00]	5.00 [1.00,20.00]	7.00 [1.50,20.00]	0.022
<=5cm	125 (46.8)	106 (51.2)	19 (31.7)	0.027
5–10cm	105 (39.3)	74 (35.7)	31 (51.7)	
>10cm	37 (13.9)	27 (13.0)	10 (16.7)	
**Multi-site resection (%)**				0.502
Yes	52 (19.5)	38 (18.4)	14 (23.3)	
No	215 (80.5)	169 (81.6)	46 (76.7)	
**Radiotherapy (%)**				0.001
yes	126 (47.2)	86 (41.5)	40 (66.7)	
no	141 (52.8)	121 (58.5)	20 (33.3)	

### Patient Outcomes and Risk Factors Associated With RFS

Among the 267 patients, 53 patients (19.85%) experienced recurrence, among which 31 patients experienced their first recurrence (31/207) and 22 patients experienced a second recurrence (22/60). The 3-year and 5-year RFS rates were 82.5% and 78%, respectively, with a median recurrence time of 15 months (range, 1–96 months).

In the Kaplan-Meier analysis, younger age, larger tumor diameter, multiple tumors, extremity tumors and recurrent tumors were significantly associated with lower RFS (**Figures 1B–F**).

**Figure 2:**
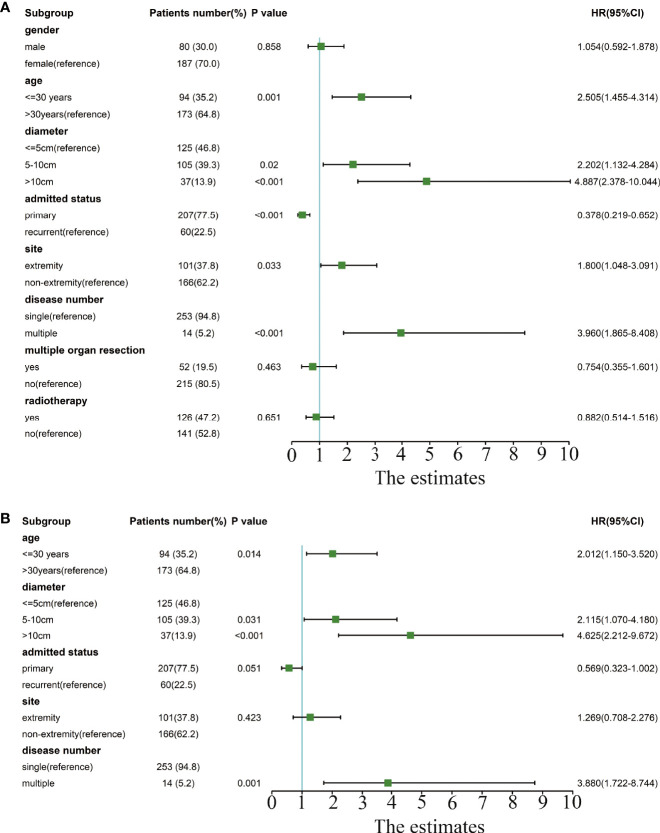
Forest plot of univariate and multivariate Cox analyses of risk factors related to recurrence. **(A)** The forest plot for univariate Cox analysis. **(B)** The forest plot for multivariate Cox analysis.

Univariate Cox analysis identified that age, tumor diameter, admission status, tumor location, and tumor number are related to recurrence, while gender, multisite combined resection, and radiotherapy are not related to recurrence (**Figure 2A**).

In the multivariate Cox analysis, only age, tumor diameter, and tumor number are independent risk factors for recurrence, while admission status and location are not related to recurrence. Specifically, patients with age<=30 years old, a tumor diameter of 5–10 cm or a tumor diameter>10 cm, and multiple tumors have a higher recurrence rate. (**Figure 2B**).

We also examined the recurrence rates of these three groups with lower RFS. The results showed that patients who were younger than 30 years old, with a tumor diameter of 5–10 cm or a tumor diameter> 10 cm, and multiple tumors had a significantly higher recurrence rate (**Table 2**).

**Table 2:** Comparison of recurrence rate in terms of three variables: age, diameter and tumor number.

Group	Patients number	Recurrent patients number	Recurrent rate	χ^2^ value	P value
**Age**				13.273	<0.001
<=30 years old	94	30	31.9%		
>30 years old	173	23	13.3%		
**Diameter**				18.884	<0.001
<=5cm	125	14	11.2%		
5-10cm	105	23	21.9%		
>10cm	37	16	43.2%		
**Tumor number**				10.560	0.001
single	253	45	17.8%		
multiple	14	8	57.1%		

### Development, Evaluation, Validation, and Visualization of the Nomogram

**Figure 3:**
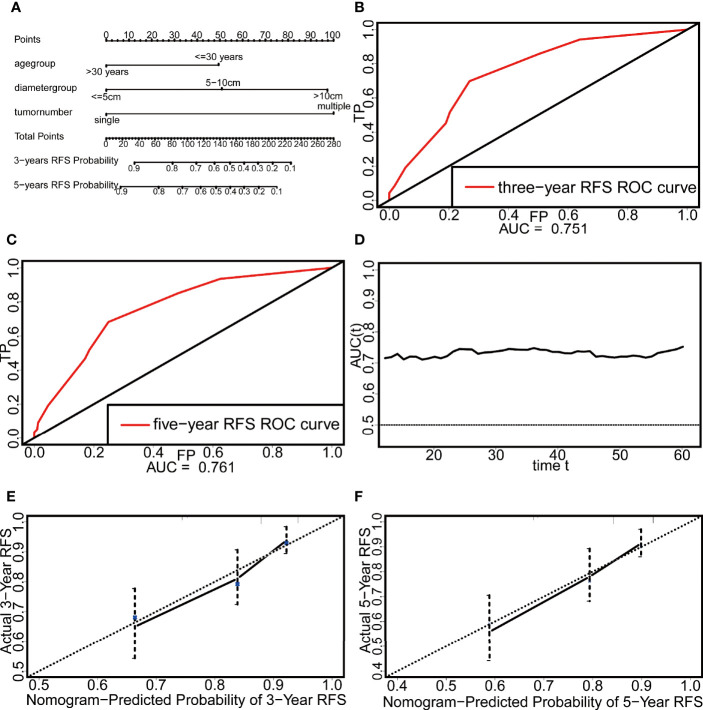
The nomogram and the evaluation of its discrimination and calibration. **(A)** The nomogram based on Cox multivariate regression. **(B)** The ROC curve of three-year RFS. **(C)** The ROC curve of five-year RFS. **(D)** The time-dependent ROC curve. **(E)** The calibration plot of three-year RFS. **(F)** The calibration plot of five-year RFS.

Three independent risk factors, namely, age, tumor diameter, and tumor number, were screened from the results of the multivariate Cox analysis. Based on these three factors, a nomogram for predicting the 3-year and 5-year RFS probabilities of patients with DTs was constructed (**Figure 3A**).

The discrimination of the nomogram was evaluated by the C-index and ROCcurve. The C-index reached 0.718, and the areas under the curve (AUCs) of the 3-year and 5-year ROC curves were 0.751 and 0.761, respectively (**Figures 3B, C**). In addition, the AUC of this nomogram after 12 months was above 0.7, which indicates that the nomogram had good discrimination (**Figure 3D**). The calibration was evaluated by the calibration plot and showed that the nomogram had good calibration (**Figures 3E, F**).

**Figure 4:**
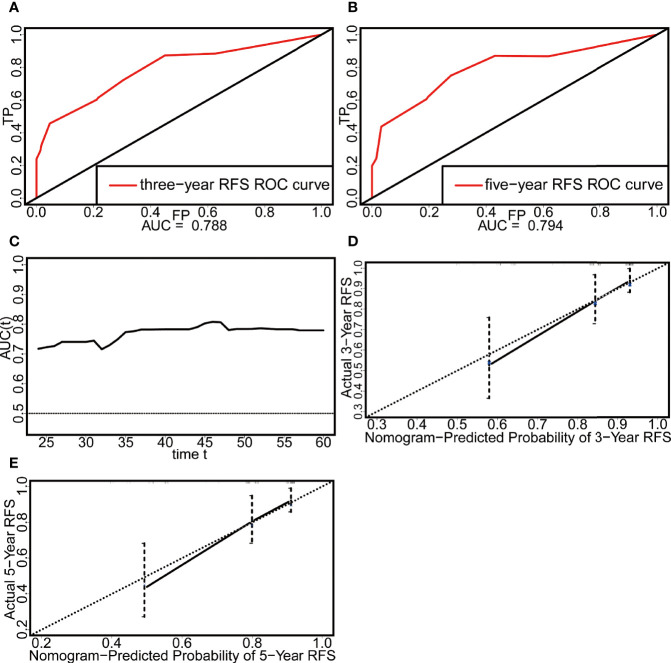
Evaluation of the discrimination and calibration of the nomogram in the external validation set. **(A)** The ROC curve of three-year RFS. **(B)** The ROC curve of five-year RFS. **(C)** The time-dependent ROC curve. **(D)** The calibration plot of three-year RFS. **(E)** The calibration plot of five-year RFS.

External validation was performed with a dataset from Fudan University Shanghai Cancer Center (n = 143) (**Table 3**). In this external validation set, the C-index reached 0.706, and the AUCs of the ROC curves at 3 and 5 years were 0.788 and 0.794, respectively (**Figures 4A, B**). Similar to that in the development dataset, the time-dependent ROC curve showed that the nomogram had good discrimination in the validation dataset (**Figure 4C**). The calibration plot also reflected the good calibration of the nomogram in the external data set (**Figures 4D, E**). However, due to the absence of DT data in the Surveillance, Epidemiology, and End Results (SEER) database and the lack of access to other databases with data on non-Chinese patients, we could not validate the nomogram in other cohorts.

**Table 3:** Patient demographics and tumor characteristics in the validation dataset.

	Overall	Primary	Recurrent	P
**Patients**	143	106 (74.1)	37 (25.9)	
**Gender (%)**				0.053
male	32 (22.4)	19 (17.9)	13 (35.1)	
female	111 (77.6)	87 (82.1)	24 (64.9)	
**Age (%)**				0.033
<=30 years	58 (40.6)	37 (34.9)	21 (56.8)	
>30 years	85 (59.4)	69 (65.1)	16 (43.2)	
**Tumor number (%)**				<0.001
Single	132 (92.3)	104 (98.1)	28 (75.7)	
Multiple	11 (7.7)	2 (1.9)	9 (24.3)	
**Diameter**				0.046
<=5cm	70 (49.0)	58 (54.7)	12 (32.4)	
5-10cm	51 (35.7)	35 (33.0)	16 (43.2)	
>10cm	22 (15.4)	13 (12.3)	9 (24.3)	
**Radiotherapy (%)**				0.007
yes	119 (83.2)	94 (88.7)	25 (67.6)	
no	24 (16.8)	12 (11.3)	12 (32.4)	

**Figure 5:**
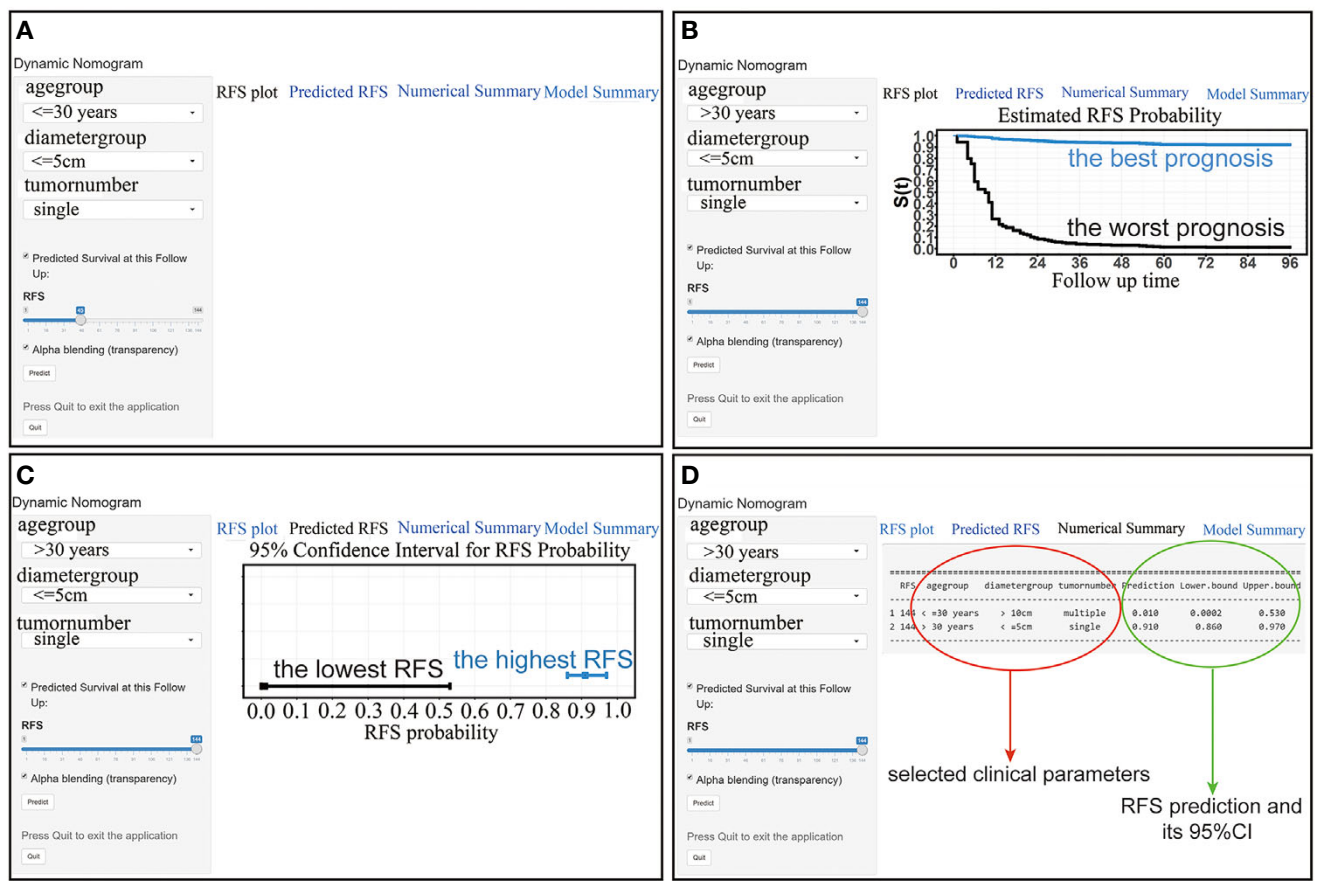
**(A)** The interface of the web-based nomogram. **(B)** RFS curves for patients with the best (blue line) and worst (black line) prognosis. **(C)** The RFS probability and its 95% CI for patients with the best (blue line) and worst (black line) prognosis. **(D)** Numerical summary of the RFS probability.

In addition, we further visualized the nomogram and created a web version (https://stepforward.shinyapps.io/Desmoidtumor/). The RFS curve and probability of the patient can be displayed by selecting the corresponding clinical features and follow-up time on the left side of the web interface (**Figure 5A**). For instance, patients with the following characteristics had the worst prognosis: age <=30 years, tumor diameter >10 cm, and multiple tumors (**Figure 5B**, black line). Patients with the following characteristics had the best prognosis: age >30 years, tumor diameter <=5 cm, and single tumors (**Figure 5B**, blue line). Additionally, the predicted RFS and its 95% CI are displayed (**Figure 5C**) as well as a specific numerical summary to make the prediction more accurate (**Figure 5D**).

From the above results, it is clear that this web-based nomogram has good discrimination and calibration in both the development population and the validation population. Furthermore, compared with traditional nomograms that can only roughly predict the recurrence probability, this web-based nomogram not only predicts the probability more accurately but also shows the 95% CI and RFS curve after the user selects several parameters on the web page, thus further reflecting the patient’s prognosis.

### Additional Analysis of Patients Who Were Lost To Follow-Up

In addition, considering that 80 patients were lost to follow-up, we attempted to include these lost patients as recurrent patients for additional analysis (**Supplemental Table 1** and **Supplemental Table 2**). The results were somewhat different from the previous results that excluded patients who were lost to follow-up. Univariate Cox analyses showed that tumor location is not related to recurrence. This result is very different from the results reported in previous studies. In three studies involving more than 400 patients, both univariate and multivariate Cox analyses showed tumor location was associated with recurrence (10, 21, 22). In several other studies that included a smaller number of patients, univariate Cox analysis showed tumor location was associated with recurrence, although multivariate Cox analysis showed no association between location and recurrence (23, 24). The reason may be that if the patients who are lost to follow-up are regarded as recurrent patients, some of them may not actually recur, resulting in bias. At the same time, we also searched other studies about desmoid tumors, and found that some studies also excluded patients who were lost to follow-up (23, 25). Therefore, it may be a reasonable approach to exclude patients who are lost to follow-up.

## Discussion

As a borderline tumor, although the mortality rate of DTs is very low, their unpredictable natural history still brings challenges to clinical treatment. The Sarcoma PAtients EuroNet (SPAEN) and European Organization for Research and Treatment of Cancer (EORTC)/Soft Tissue and Bone Sarcoma Group (STBSG) recommend active surveillance as the preferred initial treatment for DTs (26). When patients have symptoms of compression, surgery is still an important treatment, but the high recurrence rate after surgery remains a major problem for patients and doctors. Therefore, we developed and validated a nomogram and further visually integrated it into a web version for the first time. Clinicians can accurately predict the recurrence rate by selecting several parameters on the web page to strengthen postoperative monitoring and provide clinical guidance.

In contrast to the nomogram created by Crago et al. in 2013, which includes age, diameter, and location, our results show that although location was associated with recurrence in the univariate Cox analysis, the recurrence rate of tumors located in the extremity was higher than that of body tumors (p = 0.033), but location was not an independent predictor of recurrence in the multivariate Cox analysis (p = 0.423) (10). Several other studies have also found similar results in univariate and multivariate Cox analyses (23, 24). However, in two other large cohort studies, even in the multivariate Cox analysis, the conclusion that location is related to recurrence was still obtained (21, 22). Our next work will continue to collect medical records on patients with DTs in the next few years and re-analyze the relationship between tumor site and recurrence when the number of patients has further increased.

In addition, compared with the nomogram created by Crago et al. in 2013, we believe our web-based nomogram has two innovations as follows. First, we added the tumor number as a predictor for the first time, and indicated that the recurrence rate of patients with multiple tumors was higher than that of patients with single tumors (p = 0.001). Actually, multiple DTs usually occur in FAP-related patients but are rare in sporadic DTs (2). At present, the proportion of patients with multiple DTs reported in most of the literature is less than 10%. James et al. found 5 patients with multiple DTs among 101 patients, accounting for 4.95% (17). Marco et al. found 6 patients with multiple DTs out of 92 patients, accounting for 6.5% (8). Huang et al. found 14 patients with multiple DTs among 151 patients, accounting for 9.3% (27). Salas et al. found 10 patients with multiple DTs among 426 patients, accounting for 2.3% (21). Among them, only Huang et al. discussed the relationship between the tumor number and postoperative recurrence and indicated that the tumor number was not related to postoperative recurrence (p = 0.422). Two other studies with more than 400 patients also did not study a relationship between tumor number and recurrence (10, 22). We speculate that the relationship between tumor number and recurrence has rarely been studied before because multiple DTs are rare. In our study, the proportion of patients with multiple DTs was only 5.2%, which was approximately the same as that reported in other literature. Our next work is to cooperate with other hospitals to collect data from patients with multiple DTs to further confirm our conclusion. Given the rarity of multiple DTs, the association between tumor number and recurrence needs to be further confirmed in other large studies. Second, in terms of the nomogram constructed by Crago et al., patients and doctors need to find the corresponding score of each variable and add these scores to calculate a total score. Then, the probability of recurrence is predicted based on this total score. However, the steps are relatively cumbersome, and the result is only an approximate probability, without an overall understanding of the prognosis. The web-based nomogram we constructed is more visual than the traditional nomogram, eliminating the cumbersome steps of calculating scores. The web-based nomogram can directly display an accurate recurrence probability after selecting the corresponding clinical parameters and follow-up time directly on the web page and can also display a 95% CI. On the other hand, after selecting the corresponding parameters, the RFS curve can also be displayed, giving a clear understanding of the overall prognosis of the patient. Therefore, this web-based nomogram is highly visualized, accurate and intuitive.

In this study, adjuvant radiotherapy did not reduce the postoperative recurrence rate. In fact, the role of postoperative adjuvant radiotherapy has been controversial in the past few decades. Different studies have come to different conclusions (28–31). A meta-analysis has indicated that adjuvant radiotherapy can reduce the recurrence rate of patients with positive resection margins, but for patients with negative resection margins, adjuvant radiotherapy cannot reduce recurrence or improve the local control rate (32). This may also be the reason why we did not obtain the conclusion that adjuvant radiotherapy reduces recurrence in our study, as all of these patients had a negative margin status (R0) in our study. For the Chinese population, most studies still report that postoperative adjuvant radiotherapy cannot reduce recurrence (33, 34). Among them, the study of Huang et al. included 214 patients (24). Therefore, the significance of adjuvant radiotherapy remains to be further studied.

In terms of clinical application, we believe that the main application value of this web-based nomogram lies in the decision-making before surgery and enhancement of postoperative surveillance. First, for patients with a relatively high recurrence rate, such as patients aged <=30 years with a tumor diameter >10 cm and multiple tumors, the web-based nomogram indicated that the 5-year RFS rate was only 16%, suggesting that such patients are extremely prone to postoperative recurrence. In this case, surgery is not the preferred treatment, and doctors or patients can choose other treatments such as radiotherapy alone or systemic drug treatment. For patients with a low recurrence rate, such as those aged >30 years old with a tumor diameter <=5 cm and a single tumor, the web-based nomogram indicated that the 5-year RFS rate was 92%, which indicates that the probability of postoperative recurrence is low, and surgical treatment has shown its superiority compared with other treatments. Intensive follow-up is all that is needed after surgery. It is important to point out that this is a nomogram that has only been tested on patients undergoing surgery, and factors related to recurrence existed before surgery. Through this nomogram, the effectiveness and success rate of the operation can be predicted. For patients with a high recurrence rate after surgery, other treatments can be chosen. Therefore, the greater clinical significance of this nomogram lies in predicting the efficacy of surgery and the choice of treatment methods. Second, this web-based nomogram will also help enhance postoperative surveillance. For patients who have received surgical treatment, this web-based nomogram can accurately predict the recurrence probability at each time point after surgery so that doctors and patients can have a clearer understanding of the prognosis and strengthen postoperative surveillance. According to the latest Desmoid Tumor Management Guidelines published by “The Desmoid Tumor Working Group” and NCCN (Soft tissue sarcoma, 2020, V2) guidelines, DT can be followed by MRI or CT, especially MR I (12). Postoperative MRI images of recurrent DT mainly showed enhancement of T2-weighted, T2-FS and STIR sequence signals, accompanied by the enhancement of nodular or mass‐like T1-weighted postcontrast sequence signals (35, 36).

The limitations of this study cannot be ignored. First, the type of CTNNB1 gene mutation was not included in our study, and several previous studies have confirmed that the type of CTNNB1 gene mutation is also associated with the recurrence of desmoid fibromatosis. There are three types of mutations in the CTNNB1 gene, namely, T41A, S45F, and S45P, among which patients with the S45F mutation have the highest recurrence rate (1, 37, 38). We have tried to find the gross specimens of these patients from our hospital’s tissue sample bank to detect the mutation type of the CTNNB1 gene. Unfortunately, despite our best efforts, we were able to find only a few samples, which were insufficient to study the effect of the mutation type on recurrence. In our ongoing work, we will focus on collecting the gross specimens of patients to detect the mutation type of the CTNNB1 gene in patients and predict RFS more accurately. Second, 80 patients were lost to follow-up out of a total of 385 reviewed patients, and these lost cases may bias the final outcome. Third, this study is a retrospective study, which may produce bias. A prospective investigation would better enhance and validate these results. Fourth, we could not find an available non-Chinese patient cohort to validate the nomogram, even though we validated it on a dataset from another Chinese cancer center. Nevertheless, our findings could still benefit clinical practice because the web-based nomogram can strengthen postoperative monitoring.

In summary, we developed, validated, and visualized a web-based nomogram including age, tumor diameter, and tumor number based on independent risk factors associated with recurrence to predict RFS in patients with DTs. In the future, the use of multiple technologies to further search for biological or clinical markers that predict the recurrence of DTs is a development direction of surgery, such as the combination of radiomics and artificial intelligence to find the imaging characteristics of DT recurrence. The preoperative evaluation of these characteristics can further predict the risk of recurrence to determine the optimal treatment plan.

## Data Availability Statement

The raw data supporting the conclusions of this article will be made available by the authors, without undue reservation.

## Ethics Statement

The studies involving human participants were reviewed and approved by Tianjin Medical University Cancer Institute & Hospital ethics committee and performed according to the Helsinki declaration. Written informed consent for participation was not required for this study in accordance with the national legislation and the institutional requirements.

## Author Contributions

HL drafted the manuscript. KH and YC provided part of data and revised the manuscript. TL, TY, ZL, CZ, LX, YC, and JY revised the manuscript. All authors contributed to the article and approved the submitted version.

## Funding

This work was supported by the Key Nature Science Foundation of Tianjin [18YFZCSY00550 to JY].

## Conflict of Interest

The authors declare that the research was conducted in the absence of any commercial or financial relationships that could be construed as a potential conflict of interest.

## Abbreviations

AUC, areas under the curve; CI, confidence interval; DT, desmoid tumor; HR, hazard ratio; RFS, recurrence free survival; ROC, receiver operating characteristic; SEER, Surveillance, Epidemiology, and End Results.
