# Corrigendum: Development, Validation, and Visualization of A Web-Based Nomogram for Predicting the Recurrence-Free Survival Rate of Patients With Desmoid Tumors

**DOI:** 10.3389/fonc.2021.688620

**Published:** 2021-04-20

**Authors:** Haotian Liu, Kai Huang, Tao Li, Tielong Yang, Zhichao Liao, Chao Zhang, Lijie Xiang, Yong Chen, Jilong Yang

**Affiliations:** ^1^ Department of Bone and Soft Tissue Tumor, Tianjin Medical University Cancer Institute & Hospital, Tianjin, China; ^2^ National Clinical Research Center for Cancer, Key Laboratory of Cancer Prevention and Therapy, Tianjin’s Clinical Research Center for Cancer, Tianjin Medical University Cancer Institute & Hospital, Tianjin, China; ^3^ Department of Musculoskeletal Oncology, Fudan University Shanghai Cancer Center, Shanghai, China; ^4^ Department of Oncology, Shanghai Medical College, Fudan University, Shanghai, China; ^5^ Brandon Regional Hospital GME, HCA Healthcare/USF Morsani College of Medicine, Brandon, FL, United States; ^6^ Department of Bone and Soft-Tissue Tumor, Institute of Cancer and Basic Medicine, Chinese Academy of Sciences, Cancer Hospital of the University of Chinese Academy of Sciences, Zhejiang Cancer Hospital, Hangzhou, China

**Keywords:** desmoid tumor, recurrence, prediction, the web-based nomogram, tumor number, validation

In the original article, there was an error in the **Results** section of the **Abstract**: “Among these patients, 53 experienced recurrence, with a recurrence rate of 20.15%”.

A correction has been made to the **Results** section of the **Abstract**: “Among these patients, 53 experienced recurrence, with a recurrence rate of 19.85%”.

In the original article, there was an error in **Patient Outcomes and Risk Factors Associated With RFS** of the **Results**: “Among the 267 patients, 53 patients (20.15%) experienced recurrence”.

A correction has been made to **Results**, *Patient Outcomes and Risk Factors Associated With RFS*, Paragraph 1: “Among the 267 patients, 53 patients (19.85%) experienced recurrence”.

The authors apologize for these errors and state that these do not change the scientific conclusions of the article in any way. The original article has been updated.

