# Supplementary material for: Development, Validation, and Visualization of A Web-Based Nomogram for Predicting the Recurrence-Free Survival Rate of Patients With Desmoid Tumors
Source: Front Oncol. 2021 Feb 25;11:634648. doi: 10.3389/fonc.2021.634648 (PMC7947817; doi:10.3389/fonc.2021.634648)
Supplement: Supplementary file 1 [file Table_1.docx]

Supplemental Table 1 Univariate analysis of risk factors associated with recurrence

| Subgroup | P | HR | 95%CI | Comparison Variable |
| --- | --- | --- | --- | --- |
| sex |  |  |  | female |
| male | 0.599 | 0.903 | 0.617-1.322 |  |
| age |  |  |  | >30 years |
| <=30 years | 0.015 | 1.530 | 1.086-2.157 |  |
| diameter |  |  |  | <=5 cm |
| 5-10 cm | 0.015 | 1.600 | 1.096-2.335 |  |
| >10 cm | <0.001 | 2.768 | 1.678-4.541 |  |
| site |  |  |  | non-extremity |
| extremity | 0.981 | 0.996 | 0.703-1.410 |  |
| tumor number |  |  |  | single |
| multiple | 0.001 | 2.858 | 1.530-5.338 |  |
| radiotherapy |  |  |  | no |
| yes | 0.020 | 0.648 | 0.449-0.935 |  |
