# Supplementary material for: Development, Validation, and Visualization of A Web-Based Nomogram for Predicting the Recurrence-Free Survival Rate of Patients With Desmoid Tumors
Source: Front Oncol. 2021 Feb 25;11:634648. doi: 10.3389/fonc.2021.634648 (PMC7947817; doi:10.3389/fonc.2021.634648)
Supplement: Supplementary file 2 [file Table_2.docx]

Supplemental Table 2 Multivariate analysis of risk factors associated with recurrence

| Subgroup | P | HR | 95%CI | Comparison Variable |
| --- | --- | --- | --- | --- |
| age |  |  |  | >30 years |
| <=30 years | 0.026 | 1.476 | 1.047-2.081 |  |
| diameter |  |  |  | <=5 cm |
| 5-10 cm | 0.007 | 1.681 | 1.150-2.458 |  |
| >10 cm | <0.001 | 2.841 | 1.728-4.671 |  |
| tumor number |  |  |  | single |
| multiple | <0.001 | 3.273 | 1.742-6.149 |  |
| radiotherapy |  |  |  | no |
| yes | 0.010 | 0.615 | 0.425-0.889 |  |
